# Somatostatin Receptor Targeted PET-Imaging for Diagnosis, Radiotherapy Planning and Theranostics of Meningiomas: A Systematic Review of the Literature

**DOI:** 10.3390/diagnostics12071666

**Published:** 2022-07-08

**Authors:** Luca Filippi, Isabella Palumbo, Oreste Bagni, Orazio Schillaci, Cynthia Aristei, Barbara Palumbo

**Affiliations:** 1 Nuclear Medicine Unit, “Santa Maria Goretti” Hospital, Via Antonio Canova, 04100 Latina, Italy; o.bagni@ausl.latina.it; 2 Section of Radiation Oncology, Department of Medicine and Surgery, University of Perugia, Piazza Lucio Severi 1, 06132 Perugia, Italy; isabella.palumbo@unipg.it (I.P.); cynthia.aristei@unipg.it (C.A.); 3 Department of Biomedicine and Prevention, University Tor Vergata, Viale Oxford 81, 00133 Rome, Italy; orazio.schillaci@uniroma2.it; 4 IRCCS Neuromed, 86077 Pozzilli, Italy; 5 Section of Nuclear Medicine and Health Physics, Department of Medicine and Surgery, Università Degli Studi di Perugia, Piazza Lucio Severi 1, 06132 Perugia, Italy; barbara.palumbo@unipg.it

**Keywords:** meningioma, neuro-oncology, PET/CT, PET/MRI, ^68^Ga-DOTA-peptides, somatostatin receptors, radiotherapy planning, gross tumor volume, Gamma Knife, theranostics

## Abstract

The aims of the present systematic review are to: (1) assess the diagnostic performance of somatostatin receptor (SSR)targeted positron emission tomography (PET) with different tracers and devices in patients affected by meningiomas; and (2) to evaluate the theranostic applications of peptide receptor radionuclide therapy (PRRT) in meningiomas. A systematic literature search according to PRISMA criteria was made by using two main databases. Only studies published from 2011 up to March 2022 in the English language with ≥10 enrolled patients were selected. Following our research strategy, 17 studies were included for the assessment. Fourteen studies encompassed 534 patients, harboring 733 meningiomas, submitted to SSR-targeted PET/CT (*n* = 10) or PET/MRI (*n* = 4) for de novo diagnosis, recurrence detection, or radiation therapy (RT) planning (endpoint 1), while 3 studies included 69 patients with therapy-refractory meningiomas submitted to PRRT (endpoint 2). A relevant variation in methodology was registered among diagnostic studies, since only a minority of them reported histopathology as a reference standard. PET, especially when performed through PET/MRI, resulted particularly useful for the detection of meningiomas located in the skull base (SB) or next to the *falx cerebri*, significantly influencing RT planning. As far as it concerns PRRT studies, stable disease was obtained in the 66.6% of the treated patients, being grade 1–2 hematological toxicity the most common side effect. Of note, the wide range of the administered activities, the various utilized radiopharmaceuticals (^90^Y-DOTATOC and/or ^177^Lu-DOTATATE), the lack of dosimetric studies hamper a clear definition of PRRT potential on meningiomas’ management.

## 1. Introduction

Meningiomas are the most common primary intracranial tumors, accounting for 39% of all tumors and 54.5% of non-malignant tumors according to the Central Brain Tumor Registry of the United States (CBTRUS) [1]. The World Health Organization (WHO) classified meningiomas into three different grades, on the basis of histologic findings and presence/absence of brain invasion: grade 1, grade 2 (atypical), and grade 3 (anaplastic) [2]. This classification is widely used for prognostic stratification and guiding therapeutic management, although emerging evidence suggests that, aside from classical WHO grading, molecular profile should be taken into account for a more accurate and patient’s tailored clinical management [3].

Progressive meningiomas’ enlargement or compression of neighboring neural tissue may lead to neurological symptoms (i.e., generalized or focal seizures, neurological deficits), thus guiding clinicians to diagnosis. Brain magnetic resonance imaging (MRI) represents the gold standard due to its high accuracy, especially when diffusion weighted imaging (DWI) is utilized [4]. When feasible, surgery should be considered as the therapy of choice for meningiomas’ treatment, while radiation therapy (RT) is generally recommended in case of inaccessible locations and in the adjuvant setting after surgery for grade 2–3 lesions [5]. Grade 1 meningiomas are slow-growing and characterized by a good prognosis; on the contrary, grade 2–3 meningiomas present a less favorable outcome, due to their propensity to recur after surgical exeresis [6].

Somatostatin receptors (SSRs), subdivided in five different types (SSR1-5), represent a well-known class of molecules belonging to the family of 7-transmembrane G-protein-coupled receptors, expressed by normal tissue and solid tumors [7]. Among SSRs, the subtype 2 (SSR2) has been found overexpressed in meningiomas and has catalyzed attention as a potential target for diagnosis and therapy.

In the past decades, scintigraphy with ^111^In-pentetreotide was utilized for the diagnosis, intraoperative detection and response assessment of SSR2 positive tumors and also proved useful for meningiomas visualization [8,9,10]. However, it has to be underlined that ^111^In-pentetetreotide scintigraphy presents several limitations, such as the poor spatial resolution and the lack of precise anatomical correlation, partially overcome by the introduction of single photon computed tomography (SPECT/CT) hybrid technology [11,12]. In recent years, 3 ^68^Ga-DOTA-peptides (i.e., ^68^Ga-DOTATOC, DOTANOC, DOTATATE), have been introduced in clinical practice for the imaging with positron emission computed tomography (PET/CT) of SSR-expressing tumors, among whom meningiomas [13,14]. PET/CT presents higher spatial resolution and sensitivity, with respect to conventional scintigraphy, also allowing the calculation of several quantitative parameters. Nevertheless, the diagnostic performance of the various available ^68^Ga-DOTA-peptides in the different diagnostic settings (i.e., de novo diagnosis, detection of recurrence, RT planning), as well as the impact of the recent technological improvements of PET-imaging (PET/MRI, digital technology, long-axial field of view PET/CT), has yet to be fully investigated [15,16,17].

Notably, the expression of SSR2 paves the ground for combining diagnosis and therapy in a unique approach (i.e., “theranostic”), consisting in the utilization of the same or similar compounds labeled with a couple of radionuclides, one emitting photons or positrons suitable for imaging and the other emitting particles for achieving an anti-tumoral effect [18]. A paradigmatic example of theranostics is represented by peptide receptor radionuclide therapy (PRRT) with ^90^Y-DOTATOC or ^177^Lu-DOTATATE, whose introduction in clinical practice has signed a cornerstone in the management of neuroendocrine tumors (NET) [19]. PRRT has been also performed as a salvage treatment of recurrent or progressive meningiomas, as recently recognized by the European Association of Neuro-oncolgy (EANO) guidelines [20]. However, despite promising preliminary reports of PRRT in meningiomas, several issues remain to be fully addressed, such as the optimal activity to be administered, the number of cycles, and the interval among the various cycles of PRRT. Furthermore, few studies are still available in international literature, although the theranostic use of combined imaging and therapy might have a potential significant role in the management of meningiomas, such as it has been showing since many years in NET [18].

To contribute to this knowledge, the aims of the present systematic review are: (1) to assess the diagnostic performance of SSR-targeted PET/CT and PET/MRI with different tracers (i.e., ^68^Ga-DOTATOC, DOTANOC and DOTATATE) in patients affected by meningiomas in different clinical settings; and (2) to evaluate the theranostic applications of PRRT (in terms of response rate) in meningiomas.

## 2. Materials and Methods

A literature search until March 2022 was performed in PubMed and Scopus databases in order to retrieve papers related to the endpoints, according to the Preferred Reporting Items for Systematic reviews and Meta-analyses (PRISMA) guidelines [21]. The terms used were: (A) “^68^Ga-DOTATOC” OR “^68^Ga-DOTANOC” OR “^68^Ga-DOTATATE” OR “^90^Y-DOTATOC” OR “^177^Lu-DOTATATE” AND (B) “meningiomas”. The following types of studies were considered: head-to-head comparative series, matched-pair studies, clinical trials, case series, prospective studies, and retrospective cohorts. Case reports, conference proceedings, editorial commentaries, interesting images, and letters to the editor were excluded. Only studies published from 2011 up to March 2022, limited to humans, in the English language with a cohort of ≥10 enrolled patients were selected. 

Two reviewers (L.F., B.P.) conducted the literature search and independently appraised each article using a standard protocol and data extraction. The reference lists of the selected studies were carefully checked to identify any additional relevant literature. 

From each study extracted data were, respectively, for endpoint 1 (imaging): type of the study (prospective, retrospective, etc.), year and location of the study, sample size, number of lesions, clinical setting, utilized tracer and administered activity, utilized device (PET/CT or PET/MRI), and standard of reference (i.e., histopathology, clinical follow-up or correlation with conventional imaging), For endpoint 2 (theranostics): type of the study (prospective, retrospective, etc.), year and location of the study, sample size, couple of theranostic radiopharmaceuticals, activity and number of therapy cycles administered, response rate, and follow-up data.

Studies with incomplete technical or clinical data were considered ineligible. In case of studies from the same group of researchers, only the report with the highest number of enrolled patients was considered.

### Quality of the Selected Papers

Selected imaging studies (endpoint 1) were analyzed using a modified version of the Critical Appraisal Skills Programme (CASP) (https://casp-uk.net/aboutus (accessed on 15 May 2022) checklist for diagnostic test studies. Critical appraisal was performed by 2 reviewers (L.F. and I.P.), and discrepancies, if any, were resolved by discussion with senior authors (B.P., O.S. and C.A.). Due to the methodological heterogeneity of the articles and the absence of a CASP checklist suitable for an appropriate assessment of the theranostic studies, the authors decided not to perform a quality evaluation of the published papers selected for endpoint 2.

## 3. Results

### 3.1. Analysis of the Evidence

**Figure 1:**
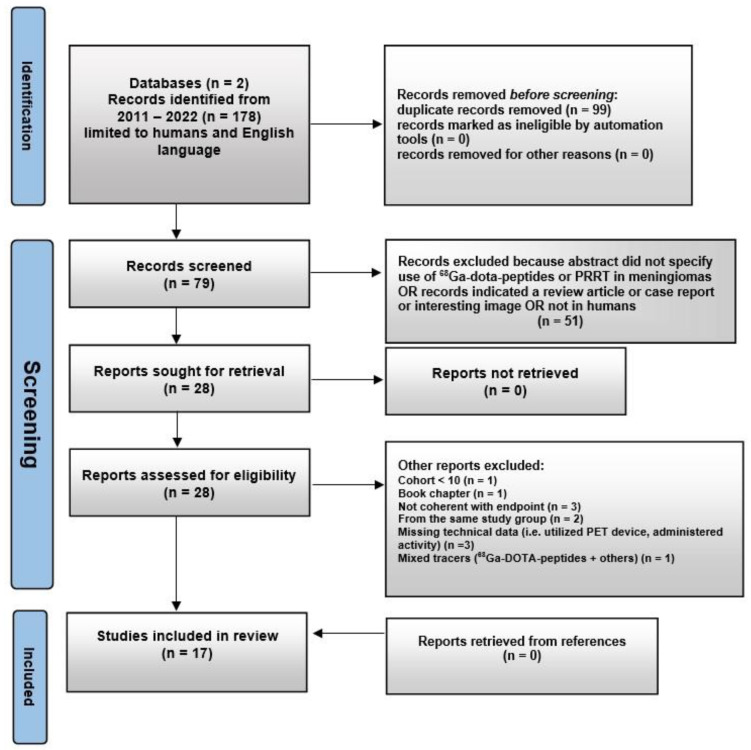
Schematic representation of PRISMA workflow for manuscripts’ selection.

**Figure 2:**
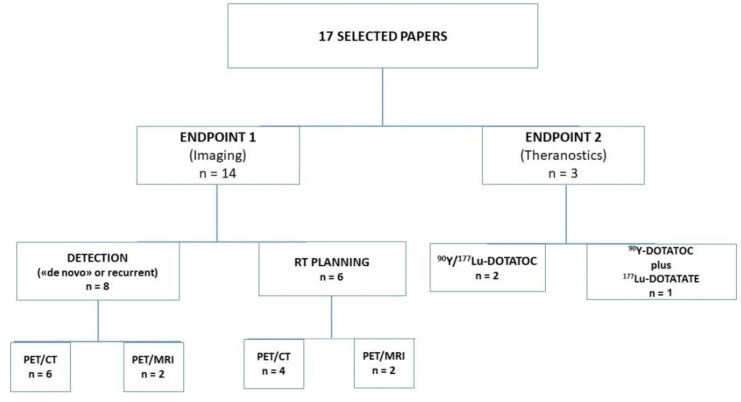
Distribution of the different selected studies for the two endpoints (imaging and theranostics). Imaging studies (**right side**) were further subdivided according to clinical settings (detection or RT planning) and technologies (PET/CT or PET/MRI), while theranostic studies (**left side**) were divided according to the radiopharmaceuticals utilized for therapeutic purposes.

The resulting PRISMA search strategy is depicted in Figure 1. From the systematic literature search, 17 papers were selected: 14 for endpoint 1, and 3 for endpoint 2. Figure 2 illustrates the distribution of the selected manuscripts according to their main endpoints and characteristics. Table 1 and Table 2 summarize the main features of the selected studies for the setting of imaging and theranostics, respectively. 

**Figure 3:**
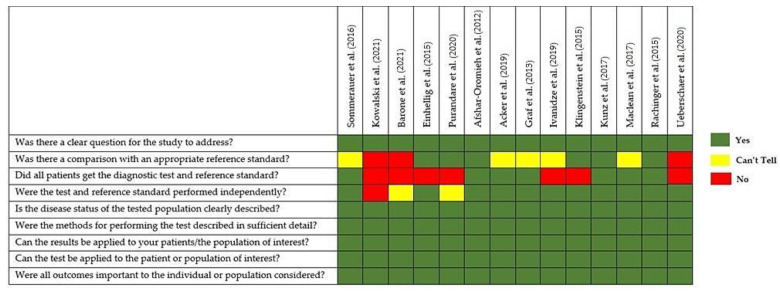
Quality appraisal of selected articles using CASP checklist for diagnostic test studies [22,23,24,25,26,27,28,29,30,31,32,33,34,35].

Based on quality assessment, diagnostic studies (endpoint 1) presented different methodologies, especially as far as it concerns the standard of reference; in fact, the majority of examined papers lacked a histological correlation or histology was not performed in all patients, as shown in Figure 3. 

### 3.2. Imaging 

SSR-targeted PET imaging was performed for meningioma diagnosis, detection of recurrence or RT planning in 14 studies, encompassing an overall number of 534 patients, harboring 733 meningiomas. Among the selected papers, 10 out of 14 (71.4%) utilized PET/CT devices, while 4 (28.6%) entailed a hybrid PET/MRI (including 1 head-to-head comparison between PET/MRI and PET/CT). As far as it concerns the different ^68^Ga-DOTA-peptides, ^68^Ga-DOTATATE was used in 8/14 studies (i.e., 57.1%), ^68^Ga-DOTATOC in 5 cases (35.7%) and ^68^Ga-DOTANOC in only 1 study (7.2%), while the administered activity ranged from 55 to 307 MBq. 

#### 3.2.1. De Novo Diagnosis and Detection of Recurrence

In a retrospective case series reported by Klingenstein et al. [24], 13 patients with symptomatic lesions of the anterior optic pathway were consecutively recruited and underwent PET/CT acquired 1 h after intravenously (i.v.) injected ^68^Ga-DOTATATE. In each case, PET/CT results were annotated in terms of lesion location and grade of uptake (i.e., SUVmax). Histopathology was utilized as a reference standard in five cases (38.5%), while in the remaining eight patients (61.5%) biopsy resulted too risky and therefore PET findings were correlated with MRI results and supplementary diagnostic work-up for clinical follow-up. ^68^Ga-DOTATATE PET/CT resulted positive in 10 cases, all considered as meningiomas at final analysis, and negative in 3 cases (then diagnosed as intracranial metastasis from gastric cancer, inflammatory connective tissue and leukemic infiltration, respectively). Although the sample size was small, PET/CT showed a sensitivity (SE) and specificity (SP) of 100%, thus being highly recommended for the characterization of ambiguous lesions of the optic pathway.

Rachinger and colleagues [25] prospectively enrolled for ^68^Ga-DOTATATE PET/CT before neuronavigated microsurgical resection a group of 21 patients with falcine/parasagittal or skull-base (SB) tumors suspected as de novo or recurrent meningioma. PET/CT scan was carried out 60 min after tracer injection, and brain MRI was also performed within a maximum time interval of 45 days: the 2 examinations were independently assessed for lesions’ location and then integrated for neuronavigated microsurgery. PET/CT showed high diagnostic accuracy both for de novo diagnosis (SE 92.3% and SP 70.0%) and for the detection of recurrent tumor (SE 88.1% and SP 76.7%), with similar specificity but higher detection rate with respect to MRI. Notably, the authors also performed histochemical analysis of SSR2 expression on tumor specimen and found a correlation between SSR2 density and tracer uptake in tumors (i.e., SUVmax), being the SUVmax threshold of 2.3 suitable for discriminating between tumor and tumor-free tissue. 

The relationship among ^68^Ga-DOTATATE meningioma uptake, considered a surrogate biomarker of SSR2 expression, and tumor growth rate (TGR) was evaluated by Sommerauer and coworkers [26] in 23 subjects with an overall number of 64 lesions. Notably, in all the enrolled patients PET/CT was started immediately after tracer administration over a time interval of 60 min; for the quantitative analysis of ^68^Ga-DOTATATE uptake (i.e., SUVmax calculation) frames acquired between 40 and 60 min were averaged. SUVmax was then correlated with TGR calculated through serial MRIs (at least 4-month interval among images). After having divided lesions into three categories (i.e., transosseous, WHO grade 1–2, WHO grade 3 meningiomas), the authors found a strong positive correlation between SUVmax and TGR for WHO grade 1 and 2 lesions (*n* = 50), being SUVmax a significant predictor of TGR in multivariate analysis. As far as it concerns transosseous meningiomas, these lesions presented higher SUVmax in comparison with the other meningiomas, with a strong correlation with TGR. WHO grade 3 meningiomas, being characterized by a lower differentiation, presented lower SUVmax and no correlation between tracer uptake and TGR. The report by Sommerauer’s group [26] is surprising, since somatostatin and its receptors generally exert an inhibitory activity on tissue growth and proliferation [7]. On the contrary, the data from the aforementioned study provide a valuable insight into the still not fully understood role of SSR2 in meningioma biology, also supporting PRRT for grade 1 and 2 lesions and for transosseus localizations, which may be particularly challenging both for surgical and RT approaches.

One of most challenging features is represented by the assessment of intracranial meningiomas’ osseous involvement, particularly for those lesions located in the SB. In this regard, Kunz and coworkers [28] performed a retrospective head-to-head comparison between ^68^Ga-DOTATATE PET/CT and contrast-enhanced MRI (ce-MRI) for the detection of meningiomas’ osseous infiltration in pre-operative and post-operative settings. PET/CT and ce-MRI were assessed by experienced readers: detected lesions were annotated for each imaging method, in terms of number and location. Furthermore, PET images were evaluated both by qualitative and quantitative analysis: for qualitative analysis, readers classified eventual osseous involvement and peri-tumoral edema through a 3-point scale, while for quantitative analysis both SUVmax and SUVmean were calculated and also lesions’ volumes were determined, via a segmentation protocol based on a 2.3 value of SUVmax as a threshold. The authors performed the comparative analysis in 82 patients with available histology as a reference standard. Among the enrolled subjects, 67 cases (i.e., 81.7%) had transosseous meningiomas, which exhibited higher SUV values and volumes with respect to extraosseous lesions. ^68^Ga-DOTATATE PET/CT presented significantly higher SE for the detection of bone infiltration than ce-MRI (i.e., 98.5% vs 53.7%, respectively), although showing a slightly lower SP (i.e., 86.7% vs. 93.3%, respectively). Notably, volumes determined by PET or MRI did not meaningfully differ among the two imaging modalities as far as it concerns the extraosseous meningiomas and for the extraosseous component of the transosseous ones, while volumes drawn by PET/CT were significantly larger for the intraosseous component of the lesions. 

In a case series including 20 patients, Ivanidze and collaborators [30] investigated the contribution of ^68^Ga-DOTATATE PET/MRI for the assessment of meningiomas, both for de novo diagnosis and for the detection of recurrence. The authors employed two distinct hybrid PET/MRI devices (i.e., Biograph mMR, Siemens Healthineers, Erlangen, Germany and GE SIGNA, GE Healthcare, Milwaukee, WI, USA), with a protocol scan starting 5–15 min after tracer i.v. administration in 3D List mode for a total time of 50 min. Images were qualitatively evaluated, by using pituitary gland as the positive reference and sagittal sinus (SSS) as the negative reference. Among the enrolled subjects, PET/MRI identified 49 lesions in 20 patients (2.5 lesions per patient). In three cases PET/MRI was helpful for the diagnosis of post-treatment changes and resulted particularly useful for defining meningiomas invasion of the osseous structures in five patients, also revealing additional lesions, not detected by conventional MRI, in four cases. Notably, quantitative analysis guided differential diagnosis between residual disease and post-treatment changes: SUVmax ratio of viable tumor/SSS, in fact, was significantly higher with respect to SUVmax ratio of post-treatment changes/SSS, although the authors did not obtain a histological confirmation to validate their findings. 

Since extent of resection (EOR) represents one of the most powerful prognostic factors on meningiomas’ outcome, Ueberschaer et al. [31] assessed the potential of PET/CT with ^68^Ga-DOTATATE for the detection of residual viable tumor tissue after neurosurgery, with respect to the traditionally applied Simpson Grade (SG), considered for many years the gold standard for the definition of the resection surgical extent [39]. SG is an intraoperative visual assessment and, although widely used in clinical practice, it has been subjected to many controversies due to its operator-dependent nature. The authors enrolled a mixed cohort, partly retrospective and partly prospective, and compared PET/CT, MRI and SG for defining EOR in resected meningiomas. PET/CT was performed 60 min after tracer administration: lesions’ number and location, as well as SUVmax, SUVmean, were evaluated. Furthermore, the authors calculated biological target volume (BTV) on PET/CT images through a segmentation algorithm based on SUVmax threshold (i.e., SUVmax = 2.3). PET/CT showed discordant findings with respect to SG both in the retrospective (i.e., 5/8 cases) and in the prospective cohort (i.e., 15/37 cases). BTV substantially differed among the two cohorts, since overall median BTV resulted in 17.1 mL in the retrospective cohort and 1.4 mL in the prospective one. Notably, 10 cases presented residual tumor tissue on PET/CT but were judged as completely resected by MRI. The majority of discrepancies were related to locations next to the convexity, SB and *falx cerebri*. Although interesting and worthy of further investigation, the report from Ueberschaer et al. [31] presents, as a major limitation, the lack of a histological verification of the data. 

Purandare and collaborators [32] applied ^68^Ga-DOTANOC PET/CT to discriminate dural metastases from meningiomas in a cohort of 42 patients with evidence of dural lesions and suspected metastasis spread from different primary tumors. The authors evaluated PET images by using a comparative 3-point scale with liver and spleen as references. Among the examined lesions, 31 were finally categorized as meningiomas: all the WHO grade 1 lesions showed high tracer uptake, while less intense tracer incorporation was observed in atypical (grade 2) and anaplastic lesions (grade 3).

In a recently published paper, Einhellig et al. [35] performed a prospective head-to-head comparison between ^68^Ga-DOTATOC PET/MRI versus standalone MRI in 57 patients harboring 112 meningiomas. Histology, as a reference standard, was available in 32 cases, while for the remaining 25, final diagnosis was made on the basis of clinical and imaging correlation. PET images were assessed both visually and quantitatively, by calculating SUV values (SUVmax-min-mean) and drawing a 3D volume including tumor. MRI detected 103 out of 112 meningiomas (91.9%), with an overall SE of 0.95 and a SP of 0.88, respectively. MRI sensitivity was higher for the lesions located in the orbit (1), with respect to those in the petroclival region (0.88). ^68^Ga-DOTATOC PET/MRI was characterized by very high SE (0.99) and SP (0.83), which resulted not meaningfully influenced by lesions’ location. Of note, SUV values were lower, although not significantly, in meningiomas missed by MRI alone.

#### 3.2.2. Radiation Therapy Planning and Post-Treatment Assessment

Afshar-Oromieh et al. [22] retrospectively investigated the diagnostic accuracy of ^68^Ga-DOTATOC PET/TC in 134 patients bearing 190 meningiomas, 82 of whom previously submitted to surgical resection and scheduled for RT. In their head-to-head comparison, the authors considered PET/CT as the index test, while MRI represented the reference standard. MRI was performed before PET/CT (with a mean time interval of 6 days) and the two examinations were independently carried out in two distinct departments. The investigators annotated the number, the location and, in case of PET/CT, the grade of uptake (SUVmax) of each detected lesions. Meningiomas diagnosed by PET/CT but located next to areas of recent surgery were excluded by the analysis, as well as those lesions suspected as meningiomas according to MRI but with no uptake at PET/CT. Afterwards, an experienced radiologist evaluated PET/CT findings to re-analyze MRI data, paying particular attention to the areas of suspected tracer uptake. Finally, the investigator assessed whether or not the irradiation field was influenced by PET/CT. Among the 134 enrolled subjects, PET/CT detected ^68^Ga-DOTATOC uptake in 190 meningiomas, while MRI disclosed 171 lesions, all positive at PET/CT scan. Therefore, an overall number of 19 out of 190 meningiomas (10%) was diagnosed by PET/CT only: MRI-missed lesions were mainly located in the petroclival region and next to the *falx cerebri*.

When planning RT, lesions located in the SB, accounting for about 30–35% of meningiomas’ overall localizations, might be particularly challenging due to their propensity to invade bone and relapse after surgery or RT [40]. In this regard, Graf et al. [23] evaluated the potential of ^68^Ga-DOTATOC PET/CT for determining gross tumor volume (GTV) in SB meningiomas scheduled for stereotactic RT, in comparison with CT and MRI. The authors examined a group of 48 patients with 54 meningiomas, 34 of whom had been previously treated with surgery or RT; as far as it concerns WHO grade, 26 were grade 1, 1 grade 2 and 27 of unknown grade. In a first step, GTV was determined on MRI and CT (GTV_MRI/CT_) and then corrected by incorporating PET information: in particular, GTV_MRI/CT_ parts with equivocal hyperintense signal at MRI without corresponding increased tracer accumulation were reviewed and eventually excluded for the determination of final GTV. At final analysis, the incorporation of PET data led to more than 10% modification of the size of final GTV in the 67% of the examined lesions. 

By virtue of its high spatial resolution and contrast imaging, hybrid PET/MRI has been successfully applied in neuro-oncology [41]. In this perspective, Maclean and colleagues [27] comparatively evaluated the impact of ^68^Ga-DOTATATE PET/MRI to reduce inter-observer variability (IOV) in defining gross target volume (GTV) with respect to conventional CT/MRI or PET/CT. The authors included meningiomas patients bearing lesions, assessed at primary or post-operative setting, whose borders resulted particularly challenging to be defined prior to RT. Enrolled subjects sequentially underwent PET/MRI and PET/CT, acquired with two distinct devices after tracer administration, both performed in the RT planning position by placing head in a thermoplastic headshell. After exams’ execution, three experienced radiation-oncologists defined tumor target volume: absolute target volumes on MRI/CT alone and MRI/CT plus either PET modality were compared. The authors reported a meaningful IOV in contouring, with higher discrepancies registered by dural tail, post-operative bed and venous sinus. Notably, PET/MRI was found to slightly improve IOV with respect to MRI/CT but not in comparison with PET/CT.

Stereotactic radiosurgery (SRS) has been applied, with satisfying clinical results, for the management of high-risk and recurrent meningiomas [42]. The precise definition of target volume as well as the determination of the boundaries of organ at risk (OARs) is of utmost importance in planning SRS. In this regard, both MRI and CT present some limitations, especially in case of SB localizations [43]. Acker and collaborators [29] assessed the impact of ^68^Ga-DOTATOC PET/MRI on the image-guided treatment planning of meningiomas scheduled for SRS with CyberKnife. Planning Target Volumes (PTVs) were defined by a highly experienced radiation oncologist (RS0) by integrating CT and PET/MRI data. Afterwards, contouring was carried out by another two radiation oncologists (RS1 and RS2), both blinded to PET data, with less experience than RS0. Furthermore, RS1 and RS2 were requested to fulfill a questionnaire assessing their subjective need for employing PET data for lesions’ contouring: both radiation oncologists expressed personally appreciation for PET/MRI with respect to CT or MRI alone, especially when lesions were located next to critical structures. The two radiation oncologists with greater experience (RS0 and RS1) delineated greater volumes than the less expert investigator (RS2). Furthermore, PTV delineated by RS1 and RS2 showed higher left-out volume in comparison with the PTV determined by RS0, who had access to PET/MRI data, thus suggesting an impact of ^68^Ga-DOTATOC PET/MRI in treatment planning of SRS for meningiomas’ therapy. 

In a case series of 19 patients, Kowalski’s group [33] investigated the usefulness of ^68^Ga-DOTATATE in meningiomas’ RT planning and post treatment evaluation. The majority of subjects were submitted to pencil beam scanning proton therapy (PBSPT): GTV was firstly delineated on CT or MRI cross-sectional imaging; afterwards, PET results were implemented in treatment planning and, if PET-positive area were identified and resulted missed by CT/MRI, RT planning was reviewed and the new lesions were eventually added. At pre-treatment phase, ^68^Ga-DOTATATE PET/CT revealed osseous involvement, not distinctly defined by MRI, in four cases with SB localization and in one case with a parietal lesion. Worthy of note, PET/CT guided diagnosis in a patient with headache and MRI-evidence of sagittal sinus thickening, previously submitted to a non-diagnostic biopsy. PET also resulted helpful for disclosing satellite lesions, missed by MRI, in three cases. Of note, PET results changed clinical management in three patients scheduled for PBSPT, being particularly helpful for the differential diagnosis between dural metastasis and meningiomas. Furthermore, PET was performed at pre- and post-treatment phase (i.e., after 3 months) in a sub-group of 10 patients. In such cases, change in total lesion activity (TLA, obtained as product of MTV and SUVmean), calculated on pre a post-RT PET scan, was compared with that obtained by MRI; a TLA decrease was registered in 9 out of 10 patients (median reduction 14.7%), while lesions’ volume resulted stable on pre/post-treatment MRI evaluation.

In a retrospective case series (*n* = 20), Barone and colleagues [34] evaluated patients with intracranial meningiomas, scheduled for Gamma Knife ICON radiosurgery, submitted to brain MRI and ^68^Ga-DOTATOC PET/CT. Furthermore, a subgroup of 12 subjects also underwent a post-treatment PET/CT scan 6 months after RT in order to gauge SUV changes. Of the enrolled patients, 18 received treatments in a single dose, while in 2 subjects bearing the largest lesions, RT was performed by administering 5 fractions. In a sub-group of 12 patients submitted to follow-up PET/CT scan, 7 cases (58%) showed a reduction in SUV value, suggestive of response. 

**Figure 4:**
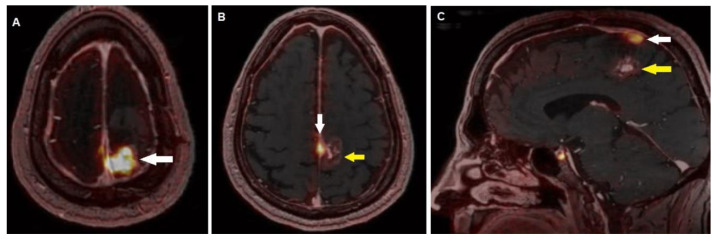
A 60-year-old male patient, previously submitted to surgery for a left parietal WHO grade III meningioma, then relapsed and treated with Gamma Knife. A 6-month post RT follow-up ^68^Ga-DOTATOC PET and contrast-enhanced T1-weighted co-registered MRI axials revealed increased tracer incorporation within the left parietal meningioma (**A**, white arrow), also detecting a focus of additional uptake in the *falx cerebri* (**B**, white arrow) adjacent to an area of “soap-bubble” contrast-enhancement of the brain parenchyma (**B**, yellow arrow), not characterized by meaningful tracer uptake, interpreted as radionecrosis. Sagittal fused PET/MRI (**C**) also well depicted the residual meningioma in the parietal region (white arrow) and the radionecrosis (yellow arrow).

An illustrative case of ^68^Ga-DOTATOC PET/MRI in a meningioma patient of our series, treated with surgery and RT (Gamma Knife) is presented in Figure 4.

### 3.3. Peptide Receptor Radionuclide Therapy 

In the selected studies for endpoint 2 [36,37,38], SSR-targeted PRRT was applied for the treatment of 69 patients affected by meningiomas recurrent or refractory to RT or surgery. ^90^Y-DOTATOC, ^177^Lu-DOTATOC, ^177^Lu-DOTATATE were utilized as a monotherapy or in various combinations, with a wide range of administered activity, for a maximum of 4 cycles at 6–8-week interval. As far as it concerns the response to PRRT, it was assessed according to MRI criteria, with 46 out of 69 patients (66.6%) having stable disease, while the remaining 23 (33.3%) presented progressive disease. In all the examined studies, renal protection was performed through the administration of amino-acid solutions containing lysine and arginine before and after each PRRT cycle to inhibit tubular reabsorption.

Marincek et al. [36] investigated the long-term outcome of PRRT in 34 patients with histologically confirmed meningiomas, progressive after surgery and/or RT within 12 months before enrollment, characterized by SSR2 expression detected through conventional ^111^In-pentetreotide scintigraphy. The authors chose ^90^Y-DOTATOC as the preferential therapeutic radiopharmaceutical for all the subjects enrolled up to 2001, then they applied a mixture of ^90^Y-DOTATOC and ^177^Lu-DOTATOC, while patients with baseline reduced renal function were treated only with ^177^Lu-DOTATOC. After the 1st cycle of PRRT, repeated cycles were performed, after a minimal interval of 6 weeks, if decreased or stable tumor size was registered or if a clinical improvement (i.e., pain relief and/or improvement of visual symptoms) was obtained. Notably, tracers’ tumor incorporation was assessed 72 h after administration through bremsstrahlung scintigraphy and the grade of uptake was evaluated on a scale ranging from score 0 (no uptake) to score 3 (uptake greater than the liver). The authors performed an overall number of 74 treatment cycles, encompassing 66 therapies with ^90^Y-DOTATOC and 8 with ^177^Lu-DOTATOC, with an administered activity ranging 1.5–18.3 GBq for the former and 7.4–22.2 GBq for the latter, respectively. Of note, the majority of enrolled patients (i.e., 41.1%) exhibited a grade 3 score. As far as it concerns response to treatment, it was assessed according to the Response Evaluation Criteria in Solid Tumors (RECIST), version 1.1 and resulted in stable disease in 23 patients (65.6%) and progressive disease in 11 cases (34.4%). During follow-up, grade 1 hematological toxicity was the most common side effect, while mean survival was 27.0 years (y) from the time of diagnosis and 8.6 y from enrollment.

In a phase II single-center study, Gerster-Gilliéron’s group [37] investigated response, survival and safety of PRRT in a cohort of 15 subjects with recurrent or progressive meningiomas, positive for SSR2 expression at ^111^In-pentetreotide scintigraphy, submitted to ^90^Y-DOTATOC with a dose fixed at 3700 MBq/m^2^ twice, with an 8-week interval. Similar to the paper from Marincek and colleagues [36], the authors assessed response to treatment 6–8 weeks after PRRT through MRI according to RECIST 1.1. Stable disease was registered in 13 patients (86.7%) and progressive disease in 2 cases (13.3%), with an overall median PFS of at least 24 months (range, 0–137 months). Treatment was well tolerated, with hematological toxicity greater than grade 2 in five cases (33.3%) and neurological toxicity in two cases (13.3%). 

Seystahl and colleagues [38] investigated the safety and effectiveness of PRRT in a cohort of 20 patients affected by progressive or therapy-refractory meningiomas and also correlated clinical outcome with pre-therapeutic expression of SSR2 assessed through ^68^Ga-DOTATATE PET/CT (*n* = 17) or ^111^In-pentetreotide scintigraphy (*n* = 3). Patients received ^177^Lu-DOTATATE in 16 cases, ^90^Y-DOTATOC in 3 and a combination of both in 1 case, with an administered activity ranging 3.4–7.6 GBq per cycle for a maximum of 4 cycles. Response to therapy was assessed through MRI or CT according to Macdonald criteria for malignant glioma [44], adapted by the authors to meningiomas, based on bi-dimensional lesions’ measurements. At post-PRRT assessment, ten subjects (50%) presented stable disease, while the remaining 10 showed progressive (50%) disease, with a median progression free survival (PFS) of 5.4 months, regardless meningiomas’ WHO grade. At statistical analysis, the authors found that pre-treatment SSR2 expression assessed by SUVmax and SUVmean by PET/CT inversely correlated with longer PFS. As reported in previously mentioned papers [36,37], therapy was generally well tolerated, with lymphocytopenia being the most frequently observed side effect.

## 4. Discussion

Meningiomas, especially when recurring or progressive after surgery or RT, represent an important issue for physicians. Although nuclear medicine techniques are not initially considered in the diagnostic process of meningiomas, the theranostic use of labeled DOTA-peptides for both diagnosis and therapy might represent a challenging option. In our review, we evaluated the available literature on this potentially emerging topic, although it is still scarce. From the careful analysis of the selected papers, some consideration about the role of SSR-targeted molecular imaging and PRRT on meningiomas’ management can be made.

Firstly, although only a limited number of the published studies have reported histology as a reference, SSR targeted PET-imaging resulted particularly useful for the detection of meningiomas, especially when located in the SB or next to the *falx cerebri*, also significantly contributing to the differential diagnosis of the lesions in the anterior optical pathways. Although different devices with different technology have been used in the available papers, it seems interesting that PET/CT has been gaining an increasing significance, showing higher SE and comparable SP with respect to brain MRI, that is generally considered the standard diagnostic modality in neuro-oncology [45]. It has to be underlined that some dural lesions, such as metastases, solitary fibrous tumors, or melanoma, although meaningfully less frequent, may mimic meningiomas and represent a diagnostic dilemma at CT and MRI [46]. Although an accurate CT and MRI assessment might aid differential diagnosis, especially through the identification of typical radiological signs (e.g., intralesional calcifications, hyperostosis, and sclerosis), it has been reported that at least 2% of resected dural lesions, initially classified as meningiomas, result in other pathologies at final histological examination [47]. Another relevant issue is represented by the differential diagnosis between meningiomas and low-grade or high-grade gliomas (LGGs/HGGs), since it has been described that they can present a similar degree of enhancement at MRI [48]. In this regard, in 50 histologically confirmed brain tumors, Zhang et al. [49] found that amide proton transfer imaging (APT) combined with conventional MRI was effective to discriminate meningiomas from LGGs/HGGs. In all the aforementioned clinical contexts, it might be reasonable to hypothesize that PET imaging with ^68^Ga-DOTA-peptides might be applied, in combination with MRI or CT, to achieve a differential diagnosis through the detection of SSR-overexpression (particularly the subtype 2), typically found in meningiomas, while lacking or only minimally detectable in the other cited pathological conditions.

Hybrid PET/MRI holds the promise to move the field of meningioma management forward, since it can combine functional imaging and PET-derived parameters with MRI’s superior 3-D soft tissue contrast obtained through T1- and T2-weighted images. Nevertheless, it has to be highlighted that, while hybrid PET/CT has been extensively applied in clinical practice with overwhelming results in oncology and non-oncological fields, the diffusion and application of PET/MRI has been hampered by several technical issues due to the presence of magnetic fields [50], therefore its potential is still not fully explored yet. 

Since RT, particularly Gamma Knife, covers a relevant role for the management of recurrent meningiomas, PET/CT and PET/MRI with ^68^Ga-DOTA-peptides proved useful for the definition of GTV, increasing inter-observer reliability with respect to CT/MRI, especially as far as the definition of the involved osseous components is concerned. In this regard, volumetric assessment and PET-derived volumetric parameters, such as MTV and TLA, proved of utmost importance for patients’ pre-treatment prognostic stratification and co-registration with CT/MRI. However, several approaches (i.e., CT-based, SUV-based, etc.) have been proposed for segmentation [51,52]; in particular, a threshold above 41% of SUVmax has been utilized for volume segmentation in neuroendocrine tumors [53]. In this regard, Kowalski and collaborators [33] applied a threshold of 2.3 SUVmax for contouring meningiomas’ lesions. Further studies are needed to better define which is the most adequate and reproducible methodology for lesions’ segmentation and volume calculation. 

^68^Ga-DOTATATE was the most utilized radiopharmaceutical for SSR targeted PET-imaging: although the different ^68^Ga-DOTA-peptides are interchangeably applied for the diagnosis and monitoring of neuroendocrine tumors in clinical practice [54], it has to be underlined that ^68^Ga-DOTATATE has been demonstrated to present a ten-fold higher affinity with respect to the other tracers [55]. However, to the best of our knowledge, a head-to-head comparison among the various ^68^Ga-DOTA-peptides for meningiomas detection, especially for the identification of small lesions in difficult locations, has not been performed yet.

A further consideration has to be made concerning the theranostic applications. A wide span between the survival data was registered in the selected studies, partially attributable to the differences in patients’ population, but also referable to the great variability of administered radiopharmaceuticals and the wide range of activities and cycles of treatments. In this regard, it has to be highlighted that ^90^Y and ^177^Lu, in spite having similar chemical characteristics, present distinct physical properties. In particular, ^177^Lu’s shorter β-particle range, with respect to ^90^Y, can entail a reduced damage to non-target tissues and a minor bone marrow toxicity [56]. Furthermore, it is worth mentioning that selected published papers on theranostics were mainly observational studies with a relatively small sample size; therefore, none of the CASP checklists for therapeutic studies (i.e., cohort/case control/randomized controlled clinical trial) resulted appropriate for a critical appraisal.

In all the analyzed papers, the various therapeutic radiotracers have been intravenously administered; in this regard, in a cohort of 4 meningioma patients Braat and coworkers [57], after having performed a 1st cycle of PRRT intravenously administered, carried out further cycles by intra-arterial ^177^Lu-HA-DOTATATE infusion: an increased target-to-background *ratio* on both planar imaging (mean, +220%) and SPECT/CT (mean, +398%) after intra-arterial administration, compared with intravenous administration, was registered. In spite of being highly promising, intra-arterial administration of therapeutic radiopharmaceuticals has still to be explored, both in terms of effectiveness and safety. 

Finally, administered activities were mainly based on empiric calculation, although in recent years, provisional dosimetry has gained a recognized role in radionuclide therapy [58]. Although dosimetric studies were not analyzed in our review, as they are not included in the aim of our work, in this regard, in a pilot trial including 11 meningioma patients treated with ^177^Lu-DOTATOC/DOTATATE, a strong correlation among tumor uptake (SUVmax) on pre-therapy ^68^Ga-DOTATOC PET/CT scan and the incorporation of therapeutic radiopharmaceutical (maximum voxel dose) during PRRT was registered [59]. 

**Figure 5:**
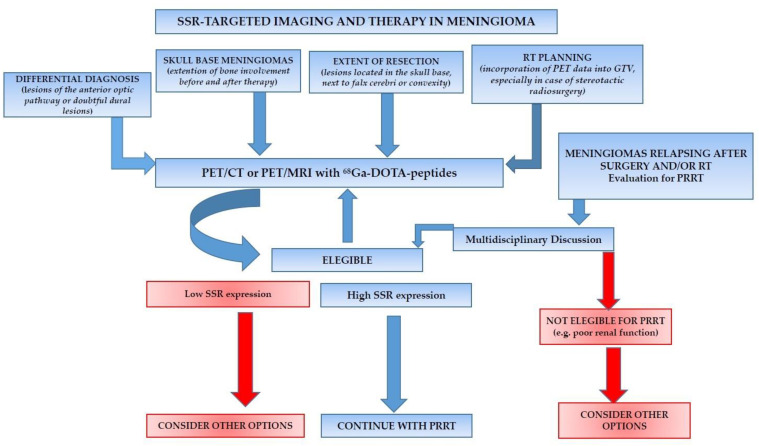
Schematic representation of the possible collocation of SSR-targeted imaging and therapy in meningiomas’ clinical work-flow, highlighting the central role of PET-imaging with ^68^Ga-DOTA-peptides.

In spite of the encouraging results reported in the selected papers, SSR-targeted imaging and therapy have not yet found a definite collocation in meningiomas’ workflow in clinical practice. Taking into account the limitations addressed in our review (e.g., lack of histological confirmation for imaging and different radiopharmaceuticals/dosages employed for the therapeutic applications), the authors believe that an SSR-based theranostics might have a role in the different settings of meningiomas’ clinical management as proposed in Figure 5. 

## 5. Conclusions

SSR-targeted PET imaging and theranostic approaches might have a great potential for meningiomas’ management, as has been widely shown in NET. However, some issues remain still to be defined. On the diagnostic side, the role of hybrid PET/MRI, especially as far as it concerns the combination of PET-derived parameters and those obtained through MRI analysis, is still broadly unexplored but appears to be particularly promising for RT planning, especially for GTV delineation and to improve inter-observer variability. On the therapeutic side, well-designed, prospective, randomized studies, including larger cohorts of patients with multi-center co-operations, are needed to define the most effective and safe therapeutic radiopharmaceutical, its dosage and administration modality. 

## Figures and Tables

**Table 1:** Analyzed manuscripts on the applications of SSR-targeted PET imaging in meningiomas.

Authors	Year	Location	Type of Study	Pts	MG	Clinical Setting	Radiotracer/Ad. Activity	Device	Reference	Comment
Afshar-Oromieh et al. [22]	2012	Germany	R H-to-H	134	190	H-to-H comparison between PET/CT and MRI for RT planning	^68^Ga-DOTATOC 139.6 MBq (55–307)	PET/CT (Biograph-6, Siemens)	MRI	PET/CT showed higher sensitivity than MRI for meningiomas’ detection, resulting particularly useful in case of location next the SB or *falx cerebri*, therefore influencing RT planning and follow-up strategy.
Graf et al. [23]	2013	Germany	R	48	54	Comparison among CT, MRI and PET for the definition of GTV in skull base meningiomas before stereotactic RT	^68^Ga-DOTATOC 70–120 MBq	PET/CT (Biograph-16, Siemens)	Comparison among imaging modalities	PET/CT led to a modification in GTV size in 32 out of the 48 examined meningiomas, thus significantly influencing RT treatment planning.
Klingenstein et al. [24]	2015	Germany	R (CS)	13	10	Role of PET for the characterization of ambiguous lesions of the optic pathways	^68^Ga-DOTATATE 210 MBq (175–254)	PET/CT (Biograph 64 TruePoint, Siemens)	Histology (*n* = 5) or follow-up	PET/CT resulted useful for correctly characterizing lesions of the anterior optic pathway and meaningfully influenced therapeutic decision.
Rachinger et al. [25]	2015	Germany	P H-to-H	21	21	H-to-H comparison between PET and MRI for detection of de novo or recurrent meningiomas	^68^Ga-DOTATATE 150 MBq	PET/CT (Biograph-64, Siemens)	Histology	PET/CT accurately identified meningiomas’ tissue both in de novo and in recurrent patients, with higher sensitivity and similar specificity with respect to MRI. Furthermore, a correlation between SSR-2 expression and SUVmax calculated on PET images was found.
Sommerauer et al. [26]	2016	Switzerland	R	23	64	Assessment of correlation among SSR expression and TGR measured by serial MRIs	^68^Ga-DOTATATE 150 MBq	PET/CT (Discovery VCT, GE Healthcare)	Follow-up	The authors found a correlation between SSR expression (measured by SUVmax) TGR in WHO grade I and II and transosseous meningiomas, limitedly to the intracranial compartment, while this correlation was not detected in WHO grade III lesions.
Maclean et al. [27]	2017	UK	P H-to-H	10	10	H-to-H comparison between PET/MRI and PET/CT for tumor contouring in meningiomas submitted to RT	^68^Ga-DOTATATE 100 MBq	PET/MRI (Biograph-mMR, Siemens) vs. PET/CT (Biograph-HiRez, Siemens)	Not applicable	PET/MRI information did not significantly improve inter-observer variability when contouring meningiomas with respect to PET/CT.
Kunz et al. [28]	2017	Germany	R H-to-H	82	82	H-to-H comparison between PET and ce-MRI for the definition of transosseous meningiomas’ extent	^68^Ga-DOTATATE 150 MBq [IQR] 129–187	PET/CT (Biograph 64 TruePoint, Siemens)	Histology	PET/CT presented higher sensitivity and specificity than ce-MRI for the detection of meningiomas’ osseous involvement. PET-based volume resulted higher than MRI-based volume in transosseous meningiomas.
Acker et al. [29]	2019	Germany	R	10	11	Influence of PET on robotic radiosurgery treatment planning	^68^Ga-DOTATOC 165 MBq; [IQR] 154–180	PET/MRI (Biograph-mMR, Siemens)	PTV	Implementation of PET/MRI data meaningfully changes PTV for robotic radiosurgery; however, the impact is strictly dependent by operator’s expertise.
Ivanidze et al. [30]	2019	USA	R (CS)	17	49	Useful of PET for pretreatment assessment, detection of recurrence, identification of additional lesions with respect to MRI	^68^Ga-DOTATATE 185 MBq	PET/MRI (Biograph-mMR, Siemens) or (SIGNA^TM^, GE Healthcare,)	Histology or follow-up	PET/MRI was useful to detect meningiomas, also revealing additional focuses with respect to conventional MRI. Furthermore, PET helped differentiating between post treatment change and tumor recurrence.
Ueberschaer et al. [31]	2020	Germany	R + P	49	52	Accuracy of PET for determining the extent of EOR vs SG	^68^Ga-DOTATATE 150 MBq	PET/CT (Biograph 64 TruePoint, Siemens)	Comparison among imaging modalities (PET/CT, MRI)	PET depicted tracer uptake indicating residual meningioma tissue after resection in 40.5% of cases classified as complete resection by neurosurgeon (SG I–II), especially in lesions next to *falx* and convexity.
Purandare et al. [32]	2020	India	R	31	31	Differential diagnosis between dural metastasis and meningiomas	^68^Ga-DOTANOC 2.64MBq/Kg	PET/CT (Philips Astonish TF systems)	Histology or Follow-up	PET/CT proved capable to discriminate dural metastasis from meningiomas, taking into account the different expression of SSR.
Kowalski et al. [33]	2021	USA	R	19	27	PET for RT planning and for (*n* = 10) post RT evaluation	^68^Ga-DOTATATE 185 MBq	PET/CT (Biograph mCT, Siemens)	MRI at 3 mo by RECIST criteria Clinical Follow-up	At pre-RT phase PET more clearly assessed tumor extent than MRI/CT and led to change in clinical management in 3 cases. In 10 pts examined pre and post-RT a decrease in PET-parameters were observed, in spite of stable MRI data.
Barone et al. [34]	2021	Italy	R (CS)	20	20	PET for planning of Gamma Knife (*n* = 12) post treatment assessment	^68^Ga-DOTATOC 110 MBq.	PET/CT (Biograph Horizon 16, Siemens) or (GE Discovery 690, GE Healthcare,)	Follow-up	PET helped identifying meningiomas before Gamma Knife therapy; in patients performing pre and post therapy assessment a decrease in SUVmax was found in the majority of cases
Einhellig et al. [35]	2021	Germany	P H-to-H	57	112	Head-to-head comparison of PET/MRI vs MRI alone for meningiomas detection	^68^Ga-DOTATOC 163.2 MBq; [IQR] 154.3–168	PET/MRI (Biograph-mMR, Siemens)	Post treatment Histology	MRI alone can detect meningiomas with high sensitivity and specificity. PET/MRI can be helpful in case of small or difficult located lesions.

Abbreviations: TGR—tumor growth rate, RT—radiation therapy, R—retrospective, CS—case series, P—prospective, H-to-H—head-to-head, min—minutes, h—hours, OBS—observational study, [IQR]—interquartile range; MG—lesions characterized by ^68^Ga-DOTA-peptides’ uptake and diagnosed as meningiomas; GTV—gross tumor volume; PTV—planning tumor volume; SB—skull base; EOR—extent of resection; SG—Simpson’s grade.

**Table 2:** Main characteristics of selected papers on PRRT applications in meningiomas.

Authors	Year	Location	Type of Study	Pts	Clinical Setting	Theranostic Couple	Ad. Activity/ N. Cycles	Response Rate	Survival OS/PFS	Follow-Up	Comment
Marincek et al. [36]	2015	Switzerland	P	34	Primary endpoint: long-term outcome	^111^In-pentetreotide/ ^90^Y-^177^Lu-DOTATOC	7.4 GBqfoi each cycle/ 1–4 cycle per patient + Renal protection	SD (*n* = 23, i.e., 65.6%) PD (*n* = 11, i.e., 34.4%)	Mean OS was 8.6 years	Mean 21.8 mo (range, 1.0–137.4 mo)	PRRT may be a useful tool for treating progressive/therapy refractory meningiomas. Stable disease after PRRT and high tracer incorporation within tumors resulted predictive of more favorable outcome.
Gerster-Gilliéron et al. [37]	2015	Switzerland	Phase II	15	Primary endpoint was survival Secondary was toxicity	^111^In-pentetreotide/ ^90^Y-DOTATOC	3700 MBq/m^2^ for 2 cycles, with an 8-wk interval + Renal protection	SD (*n* = 13, i.e., 86.7%) PD (*n* = 2, i.e., 13.3%) MRI-based at 6–8 week after therapy	Median PFS was at least 24 mo.	Mean 49.7 mo (range, 12–137 mo)	PRRT represents a feasible approach for the management of recurrent meningiomas after surgery or RT, with moderate and transient hematological toxicity.
Seystahl et al. [38]	2016	Switzerland	R	20	Safety and efficacy of PRRT in progressive meningiomas	^111^In-pentetreotide or ^68^Ga-DOTATATE-TOC/ ^90^Y-DOTATOC or ^177^Lu-DOTATATE or both	Range 3.4–7.648 GBq per each cycle/maximum 4 cycles + Renal protection	SD (*n* = 10, i.e., 50%) PD (*n* = 10, i.e., 50%), MRI-based	Median PFS was 5.4 mo, median OS not reached at follow-up	Median 20 mo (range n.a.)	PRRT in progressive meningiomas allowed disease stabilization in 50% of treated patients for a median of 17 mo. High SUVmean and low WHO grade were identified as independent prognostic factors correlated with disease control.

Abbreviations: OS—overall survival, PFS—progression free survival, wk—weeks, PRRT—peptide receptor radionuclide therapy, RT—radiotherapy, WHO—World Health Organization, mo—months, y—years;n.a.—not available.
